# An Equivalent Circuit of Longitudinal Vibration for a Piezoelectric Structure with Losses

**DOI:** 10.3390/s18040947

**Published:** 2018-03-22

**Authors:** Tao Yuan, Chaodong Li, Pingqing Fan

**Affiliations:** School of Mechatronic Engineering and Automation, Shanghai University, Shanghai 200072, China; cdli@staff.shu.edu.cn (C.L.); fanpingqing@163.com (P.F.)

**Keywords:** equivalent circuit, piezoelectric structure, loss, longitudinal vibration, impedance

## Abstract

Equivalent circuits of piezoelectric structures such as bimorphs and unimorphs conventionally focus on the bending vibration modes. However, the longitudinal vibration modes are rarely considered even though they also play a remarkable role in piezoelectric devices. Losses, especially elastic loss in the metal substrate, are also generally neglected, which leads to discrepancies compared with experiments. In this paper, a novel equivalent circuit with four kinds of losses is proposed for a beamlike piezoelectric structure under the longitudinal vibration mode. This structure consists of a slender beam as the metal substrate, and a piezoelectric patch which covers a partial length of the beam. In this approach, first, complex numbers are used to deal with four kinds of losses—elastic loss in the metal substrate, and piezoelectric, dielectric, and elastic losses in the piezoelectric patch. Next in this approach, based on Mason’s model, a new equivalent circuit is developed. Using MATLAB, impedance curves of this structure are simulated by the equivalent circuit method. Experiments are conducted and good agreements are revealed between experiments and equivalent circuit results. It is indicated that the introduction of four losses in an equivalent circuit can increase the result accuracy considerably.

## 1. Introduction

Piezoelectric structures such as bimorphs and unimorphs are of great research interest for a wide variety of applications including ultrasonic motors [1], microgrippers [2], and microcantilever biosensors [3]. Impedance or admittance curves play a remarkable role in the study of piezoelectric structures, and electromechanical properties like resonance frequency, antiresonance frequency, quality factor, and losses are revealed in those curves. Various methods are used to obtain the impedance and admittance curves theoretically. The typical one is Finite Element Analysis (FEA) which mainly relies on FEA software like ANSYS and ABAQUS, but the software is expensive and losses in piezoelectric materials are ignored. The other method is by way of a Mason equivalent circuits model. The equivalent circuit separates the piezoelectric structure into an electrical port and two acoustic ports through the use of an ideal electromechanical transformer [4]. It contains information derived from the mathematical statement of the structure, such as the piezoelectric constitutive equation and the motion equation [5], and gives much faster calculation compared with FEA [6]. Moreover, Mason’s model only uses one-dimensional assumptions and a more accurate result can be obtained [7]. Therefore, it is more suitable to use equivalent circuits to present the electromechanical behavior of piezoelectric structures. 

Mason’s equivalent circuit of piezoelectric structures has been investigated extensively since its introduction. Mason developed the Butterworth–Van Dyke (BVD) equivalent circuit from a one-port model into a three-port model by considering not only the electrical terminal but also the mechanical terminal of the piezoelectric patch [8]. Germano [9] used a simplified Mason’s equivalent circuit to describe the electromechanical and electroacoustic behaviors of a bimorph. Bao et al. [10] studied the thickness mode of a bilaminar actuator. Yang et al. [11] modeled a piezoelectric energy harvester composed of a rectangular unimorph. Wang et al. [12] simulated a bimorph piezoelectric energy harvester with segmented electrodes. The above studies focus on the bending vibration modes of piezoelectric patches and related metal substrates; thus, the motion equations which generate the equivalent circuits are of flexure vibrations of the structures, and do not take the longitudinal vibration modes into consideration because longitudinal vibrations are not the working condition of those bimorphs and unimorphs. Nevertheless, for some particular applications, such as multimode ultrasonic motors, the longitudinal vibration modes of piezoelectric patches play a remarkable role [13,14,15,16,17]. For those ultrasonic motors, first-order longitudinal vibration is used together with second- or fourth-order bending vibration to generate an elliptical motion locus at the drive feet. Except for the study of magnetoelectric laminated composites by Dong et al. [18,19], little attention has been drawn to the longitudinal vibration modes of piezoelectric structures. 

Another aspect paid little attention is the loss of metal substrates in piezoelectric structures. Loss mechanisms of piezoelectric materials have been investigated for a long time and plenty of studies have been conducted. Holland and Uchino extensively described the losses in piezoelectric materials, and found that there are three losses: dielectric, elastic, and piezoelectric losses [20,21]. Sherrit et al. [22] compared the KLM (Krimholtz, Leedom, and Matthae) and Mason’s equivalent circuits including the three losses. Chen et al. [23] established an equivalent circuit composed of complex material numbers which represent the three losses. Dong et al. [24] developed Mason’s equivalent circuits with three losses and external loads for different configurations of electrodes. Among those studies, “pure” piezoelectric ceramics are the targets when the equivalent circuits are related with losses and no metal substrates are concerned. This approach simplifies the derivation of motion equations, while for practical applications, the metal substrates are included. Therefore, it is more accurate to take the whole structure as a “composite” when investigating the properties of piezoelectric structures. In addition, since the metal substrates account for large portions of the entire structures, the elastic losses of the metal substrate should not be neglected while the losses in the piezoelectric ceramics are involved.

The aim of this paper is to establish an equivalent circuit for a beamlike piezoelectric structure in longitudinal vibration mode. In the motion equation, elastic loss in the metal substrate and three losses in the piezoelectric patch are built into the related parameters, and the longitudinal vibration mode is dealt with. Based on the equation, an equivalent circuit with four kinds of losses is derived. Using MATLAB, the impedance curve of the structure is obtained by calculating the equivalent circuit. Finally, the effectiveness of the equivalent circuit considering both elastic loss of the metal substrate and the three piezoelectric material losses is verified through experiments.

## 2. Loss and Motion Equation of the Piezoelectric Structure

**Figure 1:**
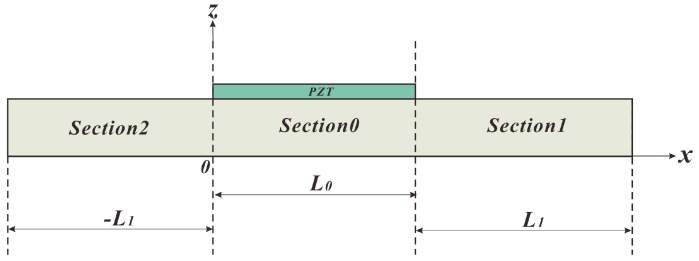
Piezoelectric structure consisting of a slender beam and a piezoelectric patch (PZT).

Figure 1 shows the configuration of the piezoelectric structure, which consists a slender beam (metal substrate, the length/thickness ratio is 15.9) made of aluminum and a piezoelectric patch (PZT). The whole structure divides into four parts; Sections 0–2 represent the metal substrate. L_0_ and L_1_ denote the lengths of related sections in Cartesian coordinates. The nomenclature used in this paper is summarized in Appendix B.

Damping, which is responsible for dissipation of energy, is one of the key properties that determines the dynamic responses of structures [25]. Damping from the oscillatory system results in the decay of amplitude of free vibration [26]. Losses expressed in complex numbers are among the most suitable indices for describing damping [27]. In this paper, we apply the superscript “*” to indicate the complex number parameters of the metal substrate and PZT. It should be noted that the superscript “*” differs from the complex conjugate. For the metal substrate, we use aluminum, and the complex Young’s modulus is defined to describe the properties of aluminum.

(1) $$Y_{M}^{*}=Y_{M}(1+j\tan\phi_{M})$$

where $Y_{M}^{*}$ is the complex Young’s modulus of aluminum, $Y_{M}$ is the Young’s modulus, *j* is the imaginary notation, and $tan\;\phi_{M}$ is the loss factor of aluminum, where the subscript “*M*” indicates the metal substrate (aluminum). 

For piezoelectric materials, heat generation due to losses leads to the degradation of material properties; losses are a major concern for miniaturized devices with high power density. Loss in piezoelectric materials is considered to have three components: dielectric, elastic, and piezoelectric. The tangent functions with superscript “′”—$tan\;\delta^{\prime }$, $tan\;\phi^{\prime }$, and $tan\;\theta^{\prime }$—are used to represent “intensive” dielectric, elastic, and piezoelectric loss factors; $tan\;\phi_{P}$ represents the “extensive” elastic loss factor and the subscript “*P*” indicates the PZT [21,28]. In this paper, we use PZT5 for its relatively large losses and low quality factor. Compared with PZT4, which has small losses and a higher quality factor, the usage of PZT5 can illustrate the great impact of PZT losses when they are considered in the equivalent circuit model.

(2) $$C_{11}^{E*}=C_{11}^{E}(1+j\tan\phi_{P})$$

(3) $$\varepsilon_{33}^{T*}=\varepsilon_{33}^{T}(1-j\tan\delta^{\prime })$$

(4) $$s_{11}^{E*}=s_{11}^{E}(1-j\tan\phi^{\prime })$$

(5) $$d_{31}^{*}=d_{31}(1-j\tan\theta^{\prime })$$

where $C_{11}^{E}$ is the stiffness under a constant electric field, $\varepsilon_{33}^{T}$ is the dielectric constant under constant stress, $s_{11}^{E}$ is the compliance under a constant electric field, and $d_{31}$ is the piezoelectric constant. 

A differential equation of motion is the basis for equivalent circuit establishment. Since this structure is a slender beam and we assume that the boundary condition is free–free, with no external force and only axial stress considered, the standard 3D piezoelectric constitutive equation can be reduced to a 1D form [29]:

(6) $$\{\begin{array}{c} D_{3}^{*}=\varepsilon_{33}^{T*}E_{3}+d_{31}^{*}X_{1}^{*}\\ S_{1}^{*}=d_{31}^{*}E_{3}+s_{11}^{E*}X_{1}^{*} \end{array}.$$

Losses in the piezoelectric patch and metal substrate are included in the derivation, so complex numbers are used and are denoted with a superscript “*”, as mentioned before. Here, *D*_3_ is the electric displacement in the *z*-direction, *E*_3_ is the electric field in the *z*-direction, *X*_1i_ is the axial stress in the *x*-direction, and *S*_1_ is the axial strain in the *x*-direction.

**Figure 2:**
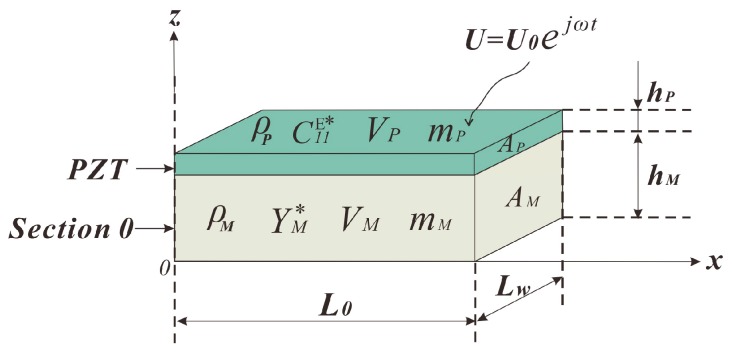
Section 0 and PZT under electric excitation and the corresponding parameters.

Section 0 and PZT in this structure play the main role and act as the excitation source; the motion equation is derived upon it and the corresponding parameters are depicted in Figure 2. Here, *ρ*, *V*, *m*, *A*, and *h* stand for the density, volume, weight, cross-sectional area, and thickness, respectively; the related subscripts “*P*” and “*M*” indicate that those parameters describe the PZT and the metal substrate (aluminum), respectively; and similarly hereinafter. A driving voltage *U* is applied on the electrode surface of the PZT, *U*_0_ is the amplitude of voltage, and *E*_3_ = *U*/*h_P_*. *L_w_* stands for the width of the structure. 

Using the Lagrange function and the variational principle [30], the motion equation of the piezoelectric structure under longitudinal vibration mode is obtained:

(7) $$\rho_{M}A_{M}(\frac{\partial^{2}u^{*}}{\partial t^{2}})+\rho_{P}A_{P}(\frac{\partial^{2}u^{*}}{\partial t^{2}})=A_{P}C_{11}^{E*}(\frac{\partial^{2}u^{*}}{\partial x^{2}})+A_{M}Y_{M}^{*}(\frac{\partial^{2}u^{*}}{\partial x^{2}})$$

where $u^{*}$ is the displacement along the *x*-direction and is a function of both position *x* and time *t*.

According to the principle of composite materials, we can rewrite Equation (7) as the following:

(8) $$\rho_{B}(\frac{\partial^{2}u^{*}}{\partial t^{2}})=Y_{B}^{*}(\frac{\partial^{2}u^{*}}{\partial x^{2}})$$

where $\rho_{B}$ is the density of the PZT and metal substrate (aluminum) as a composite material and $Y_{B}^{*}$ is the composite Young’s modulus as a complex number [31].

(9) $$\rho_{B}=\frac{m_{M}+m_{P}}{V_{M}+V_{P}}$$

(10) $$Y_{B}^{*}=\frac{V_{P}}{V_{M}+V_{P}}C_{11}^{E*}+\frac{V_{M}}{V_{M}+V_{P}}Y_{M}^{*}$$

## 3. Equivalent Circuit with Four Kinds of Losses

Equivalent circuits derive from the mathematical statement of the structure, namely, the motion equation. Although the calculation procedures of the equivalent circuit are developed in Mason’s edited book [32], the main parameters presented here are in complex numbers. For Section 0 and PZT of this piezoelectric structure, the motion equation is expressed in Equation (8). Since we focus on the longitudinal vibration, a displacement formula for an arbitrary point in Section 0 and PZT is assumed:

(11) $$u^{*}(x,t)=[\alpha \cos(k_{0}^{*}x)+\beta \sin(k_{0}^{*}x)]e^{j\omega t},$$

where *α* and *β* are coefficients that will be introduced later. Here, $k_{0}^{*}$ is the wave number rewritten as a complex number, and

(12) $$k_{0}^{*}=\omega \sqrt{\frac{\rho_{B}}{Y_{B}^{*}}}$$

where *ω* is the angular frequency. 

The velocity formula for an arbitrary point is

(13) $$\begin{array}{l} \nu^{*}=\frac{\partial u^{*}(x,t)}{\partial t}\\ \;=j\omega [\alpha \cos(k_{0}^{*}x)+\beta \sin(k_{0}^{*}x)]e^{j\omega t} \end{array}.$$

**Figure 3:**
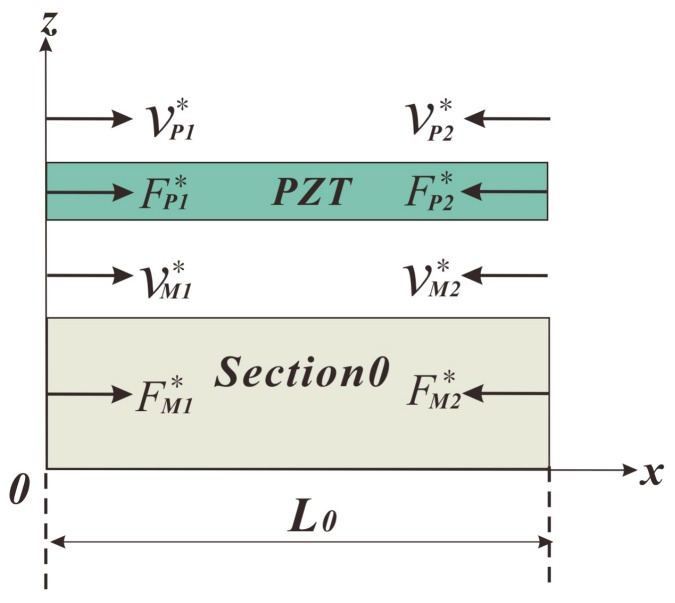
The distribution of forces and velocities in Section 0 and PZT.

The distribution of forces and velocities in Section 0 and PZT is shown in Figure 3, where *ν* stands for velocity and F for force. The subscript “*P*” represents the PZT, and “*M*” the metal substrate; the other subscript “1” means that the corresponding parameter is located at the position of *x* = 0, and “2” means the same but at *x* = *L*_0_.

### 3.1. Equivalent Circuit of PZT

First of all, we conduct the equivalent circuit of the PZT part. According to Figure 3, we have

(14) $$\{\begin{array}{c} \nu_{P1}^{*}=\nu_{P1}^{*}|{}_{x=0}\\ \nu_{P2}^{*}=-\nu_{P1}^{*}|{}_{x=L_{0}} \end{array}.$$

By inserting Equation (13) into (14) and calculating coefficients *α* and *β*, we get

(15) $$\{\begin{array}{c} \alpha =\frac{\nu_{P1}^{*}}{j\omega e^{j\omega t}}\\ \beta =-\frac{1}{j\omega e^{j\omega t}}\left[\frac{\nu_{P1}^{*}}{\tan(k_{0}^{*}L_{0})}+\frac{\nu_{P2}^{*}}{\sin(k_{0}^{*}L_{0})}\right] \end{array}.$$

The strain of an arbitrary point in the PZT is

(16) $$\begin{array}{l} S_{1}^{*}=\frac{\partial u^{*}(x,t)}{\partial x}\\ \;=-\frac{\nu_{P1}^{*}k_{0}^{*}\sin(k_{0}^{*}x)}{j\omega }-\frac{k_{0}^{*}\cos(k_{0}^{*}x)}{j\omega }[\frac{\nu_{P1}^{*}}{\tan(k_{0}^{*}L_{0})}+\frac{\nu_{P2}^{*}}{\sin(k_{0}^{*}L_{0})}]. \end{array}$$

The corresponding stress is

(17) $$\begin{array}{l} X_{1P}^{*}=\frac{1}{s_{11}^{E*}}S_{1}^{*}-\frac{d_{31}^{*}}{s_{11}^{E*}}E_{3}\\ \;=-\frac{\nu_{P1}^{*}k_{0}^{*}\sin(k_{0}^{*}x)}{j\omega s_{11}^{E*}}-\frac{k_{0}^{*}\cos(k_{0}^{*}x)}{j\omega s_{11}^{E*}}[\frac{\nu_{P1}^{*}}{\tan(k_{0}^{*}L_{0})}+\frac{\nu_{P2}^{*}}{\sin(k_{0}^{*}L_{0})}]-\frac{d_{31}^{*}}{s_{11}^{E*}}E_{3}. \end{array}$$

Then, the forces can be expressed as

(18) $$\begin{array}{l} F_{P1}^{*}=-A_{P}X_{1P}^{*}|{}_{x=0}\\ \;=\frac{A_{P}k_{0}^{*}}{j\omega s_{11}^{E*}}\left[\frac{\nu_{P1}^{*}+\nu_{P2}^{*}}{\sin(k_{0}^{*}L_{0})}-\nu_{P1}^{*}\tan(\frac{k_{0}^{*}L_{0}}{2})\right]+\frac{d_{31}^{*}}{s_{11}^{E*}}L_{w}U \end{array}$$

(19) $$\begin{array}{l} F_{P2}^{*}=-A_{P}X_{1P}^{*}|{}_{x=L_{0}}\\ \;=\frac{A_{P}k_{0}^{*}}{j\omega s_{11}^{E*}}\left[\frac{\nu_{P1}^{*}+\nu_{P2}^{*}}{\sin(k_{0}^{*}L_{0})}-\nu_{P2}^{*}\tan(\frac{k_{0}^{*}L_{0}}{2})\right]+\frac{d_{31}^{*}}{s_{11}^{E*}}L_{w}U. \end{array}$$

The following is the calculation of the current flow I*. The charge Q* of the electrode surface is

(20) $$\begin{array}{l} Q^{*}=\iint D_{3}^{*}dxdy\\ \;=\frac{d_{31}^{*}}{s_{11}^{E*}}L_{w}\left[\alpha \cos(k_{0}^{*}L_{0})+\beta \sin(k_{0}^{*}L_{0})-\alpha \right]e^{j\omega t}+\left(\varepsilon_{33}^{T*}-\frac{d_{31}^{*2}}{s_{11}^{E*}}\right)L_{w}L_{0}\frac{U_{0}e^{j\omega t}}{h_{P}} \end{array}.$$

The current flow I* is

(21) $$\begin{array}{l} I^{*}=\frac{dQ^{*}}{dt}\\ \;=-\frac{d_{31}^{*}}{s_{11}^{E*}}L_{w}\left[\nu_{P1}^{*}+\nu_{P2}^{*}\right]+j\omega \left(\varepsilon_{33}^{T*}-\frac{d_{31}^{*2}}{s_{11}^{E*}}\right)L_{w}L_{0}\frac{U_{0}e^{j\omega t}}{h_{P}} \end{array}$$

and we assume

(22) $$C_{0}^{*}=\frac{\left(\varepsilon_{33}^{T*}-\frac{d_{31}^{*2}}{s_{11}^{E*}}\right)L_{w}L_{0}}{h_{P}}$$

(23) $$N^{*}=\frac{d_{31}^{*}L_{w}}{s_{11}^{E*}}.$$

Meanwhile, we use the following equations to simplify the expression of Equations (18)–(21).

(24) $$\frac{A_{P}k_{0}^{*}}{j\omega s_{11}^{E*}}\frac{1}{\sin(k_{0}^{*}L_{0})}=R_{P1}+jG_{P1}$$

(25) $$\frac{A_{P}k_{0}^{*}}{j\omega s_{11}^{E*}}\tan(\frac{k_{0}^{*}L_{0}}{2})=R_{P2}+jG_{P2}$$

(26) $$C_{0}^{*}=C_{01}+jC_{02}$$

In Equations (24) and (25), we collect all the real parts of those parameters together and mark them as $R_{P1}$ and $R_{P2}$, which represent resistors (the mechanical energy consumption). The imaginary parts of those parameters are marked as $G_{P1}$ and $G_{P2}$, which stand for reactance (the mechanical energy storage). The complete expressions of $R_{P1}$, $R_{P2}$, $G_{P1}$, and $G_{P2}$ are listed in Appendix A. In Equation (26), the real part and imaginary part of $C_{0}^{*}$ are separated into *C*_01_ and *C*_02_. 

By inserting Equations (24)–(26) into Equations (18)–(21) correspondingly, we have the final equations to decide the equivalent circuit of PZT, as follows:

(27) $$\{\begin{array}{c} F_{P1}^{*}=(R_{P1}+jG_{P1})(\nu_{P1}^{*}+\nu_{P2}^{*})-(R_{P2}+jG_{P2})\nu_{P1}^{*}+N^{*}U\\ F_{P2}^{*}=(R_{P1}+jG_{P1})(\nu_{P1}^{*}+\nu_{P2}^{*})-(R_{P2}+jG_{P2})\nu_{P2}^{*}+N^{*}U\\ I{}^{*}=-N^{*}(\nu_{P1}^{*}+\nu_{P2}^{*})+j\omega UC_{01}-\omega UC_{02} \end{array}$$

**Figure 4:**
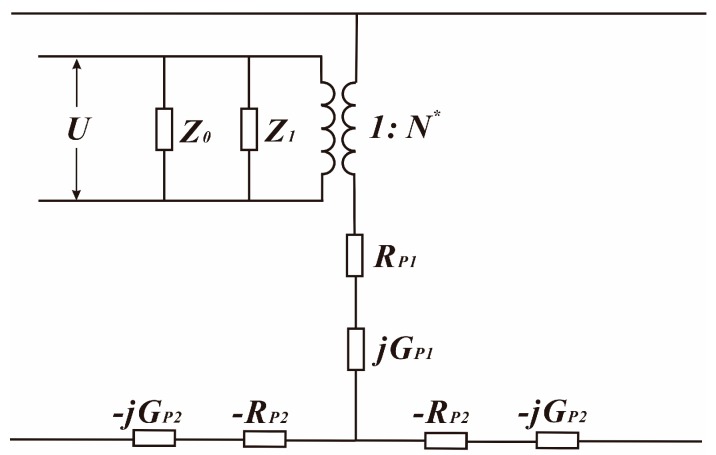
The equivalent circuit of the PZT.

Kirchhoff’s current law and Kirchhoff’s voltage law are applied to build the equivalent circuit of PZT, as shown in Figure 4, where $Z_{0}=\frac{1}{j\omega C_{01}}$, $Z_{1}=\frac{1}{-\omega C_{02}}$.

### 3.2. Equivalent Circuit of Section 0 and Complete Equivalent Circuit

According to Figure 3, and ignoring the effect of the bonding layer, we have

(28) $$\{\begin{array}{c} \nu_{M1}^{*}=\nu_{P1}^{*}\\ \nu_{M2}^{*}\;=\nu_{P2}^{*} \end{array}.$$

The strain of an arbitrary point in Section 0 is

(29) $$\begin{array}{l} S_{1}^{*}=\frac{\partial u^{*}(x,t)}{\partial x}\\ \;=-\frac{\nu_{M1}^{*}k_{0}^{*}\sin(k_{0}^{*}x)}{j\omega }-\frac{k_{0}^{*}\cos(k_{0}^{*}x)}{j\omega }[\frac{\nu_{M1}^{*}}{\tan(k_{0}^{*}L_{0})}+\frac{\nu_{M2}^{*}}{\sin(k_{0}^{*}L_{0})}]. \end{array}$$

The corresponding stress is

(30) $$\begin{array}{l} X_{1M}^{*}=Y_{B}^{*}S_{1}^{*}\\ \;=-\frac{Y_{B}^{*}\nu_{M1}^{*}k_{0}^{*}\sin(k_{0}^{*}x)}{j\omega }-\frac{Y_{B}^{*}k_{0}^{*}\cos(k_{0}^{*}x)}{j\omega }[\frac{\nu_{M1}^{*}}{\tan(k_{0}^{*}L_{0})}+\frac{\nu_{M2}^{*}}{\sin(k_{0}^{*}L_{0})}]. \end{array}$$

The forces can then be expressed as

(31) $$\begin{array}{l} F_{M1}^{*}=-A_{M}X_{1M}^{*}|{}_{x=0}\\ \;=\frac{A_{M}Y_{B}^{*}k_{0}^{*}}{j\omega }\left[\frac{\nu_{M1}^{*}+\nu_{M2}^{*}}{\sin(k_{0}^{*}L_{0})}-\nu_{M1}^{*}\tan(\frac{k_{0}^{*}L_{0}}{2})\right] \end{array}$$

(32) $$\begin{array}{l} F_{M2}^{*}=-A_{M}X_{1M}^{*}|{}_{x=L_{0}}\\ \;=\frac{A_{M}Y_{B}^{*}k_{0}^{*}}{j\omega }\left[\frac{\nu_{M1}^{*}+\nu_{M2}^{*}}{\sin(k_{0}^{*}L_{0})}-\nu_{M2}^{*}\tan(\frac{k_{0}^{*}L_{0}}{2})\right]. \end{array}$$

We use the following equations to simplify the expression of Equations (31) and (32). The complete expressions of $R_{M1}$, $R_{M2}$, $G_{M1}$, and $G_{M2}$ are listed in Appendix A and their meanings are the same as introduced before.

(33) $$\frac{A_{M}Y_{B}^{*}k_{0}^{*}}{j\omega }\frac{1}{\sin(k_{0}^{*}L_{0})}=R_{M1}+jG_{M1}$$

(34) $$\frac{A_{M}Y_{B}^{*}k_{0}^{*}}{j\omega }\tan(\frac{k_{0}^{*}L_{0}}{2})=R_{M2}+jG_{M2}$$

By inserting Equations (33) and (34) into Equations (31) and (32) correspondingly, we have the final equations to decide the equivalent circuit of Section 0, as follows:

(35) $$\{\begin{array}{c} F_{M1}^{*}=(R_{M1}+jG_{M1})(\nu_{M1}^{*}+\nu_{M2}^{*})-(R_{M2}+jG_{M2})\nu_{M1}^{*}\\ F_{M2}^{*}=(R_{M1}+jG_{M1})(\nu_{M1}^{*}+\nu_{M2}^{*})-(R_{M2}+jG_{M2})\nu_{M2}^{*} \end{array}.$$

**Figure 5:**
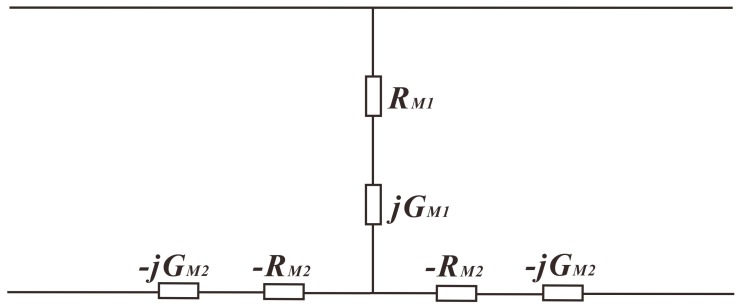
The equivalent circuit of Section 0.

Then, Kirchhoff’s current law and voltage law are applied to build the equivalent circuit of Section 0, as shown in Figure 5.

**Figure 6:**
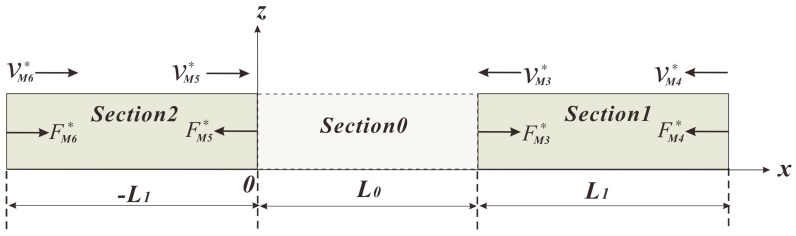
Schematic diagram of Section 1 and Section 2.

The equivalent circuits of remaining sections have the same derivation procedures as that of Section 0, apart from the different lengths and different directions of forces and velocities, as shown in Figure 6. 

**Figure 7:**
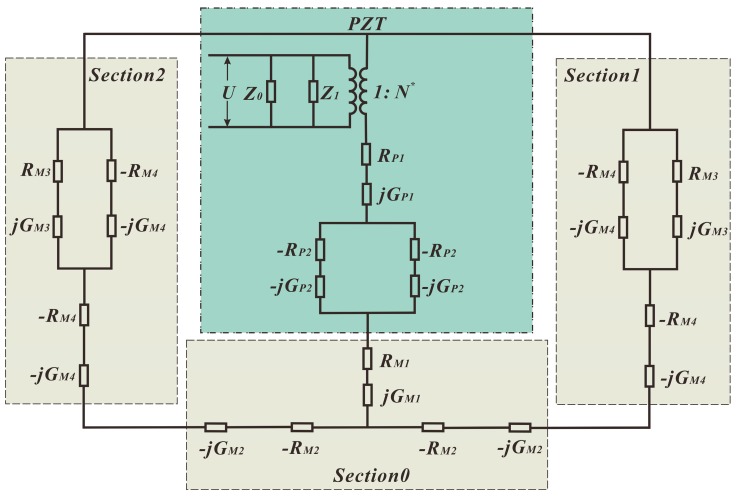
Complete equivalent circuit of the piezoelectric structure.

Finally, by combining the above separate equivalent circuits together, we can acquire the complete equivalent circuit of the introduced piezoelectric structure, as shown in Figure 7. The complete expressions of $R_{M3}$, $R_{M4}$, $G_{M3}$, and $G_{M4}$ are listed in Appendix A. This equivalent circuit represents the longitudinal vibration mode of a beam with a PZT covering part of the beam length. The principle of our new equivalent circuit differs from the conventional Mason’s equivalent circuit, for losses in the metal substrate and PZT are integrated, as mentioned in the introduction. The effectiveness of the proposed equivalent circuit will be verified through experiments.

## 4. Experiment

**Figure 8:**
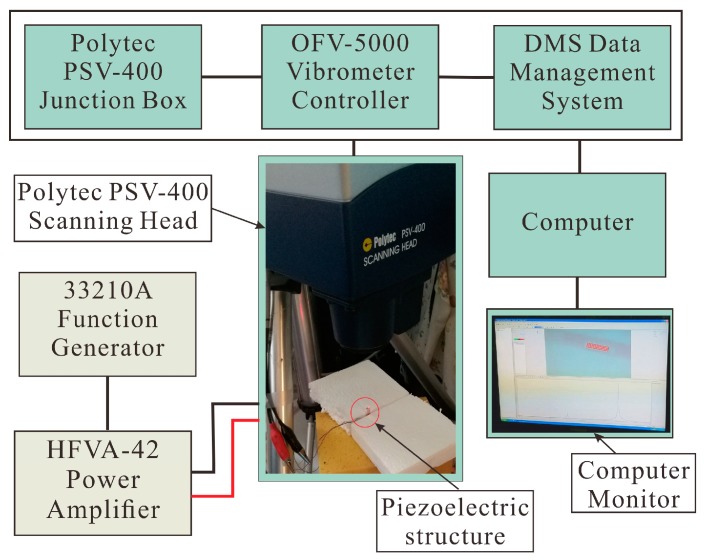
Experimental setup of vibrometer measurement.

In order to verify the effectiveness of our equivalent circuit, a prototype of this piezoelectric structure was fabricated and tested using a Polytec PSV-400 laser vibrometer (PolyTec Inc., Waldbronn, Germany). The experimental setup is shown in Figure 8. The piezoelectric structure is supposed to work at free–free boundary conditions, so it was lightly fixed using expanded polystyrene boards to avoid restriction on its longitudinal vibration. The driving signal was generated by a function generator (33210A, Keysight Technologies, Inc., Santa Rosa, CA, USA) and amplified by a power amplifier (HFVA-42, Nanjing Foneng Technology Industry Co., Ltd., Nanjing, China). The vibrometer measurement system contained a Junction Box, Vibrometer Controller, Data Management System, Scanning Head, computer, and monitor, as shown in Figure 8. 

**Figure 9:**
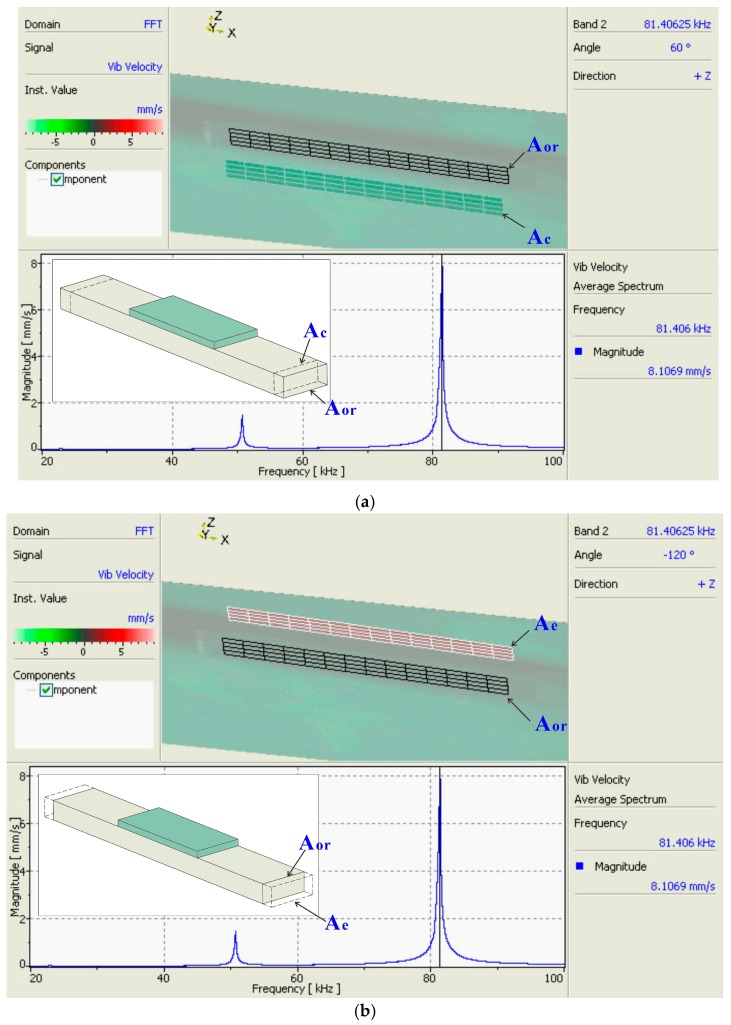
Laser vibrometer result of piezoelectric structure under longitudinal vibration mode: (**a**) longitudinal vibration under contraction situation; (**b**) longitudinal vibration under extension situation.

**Figure 10:**
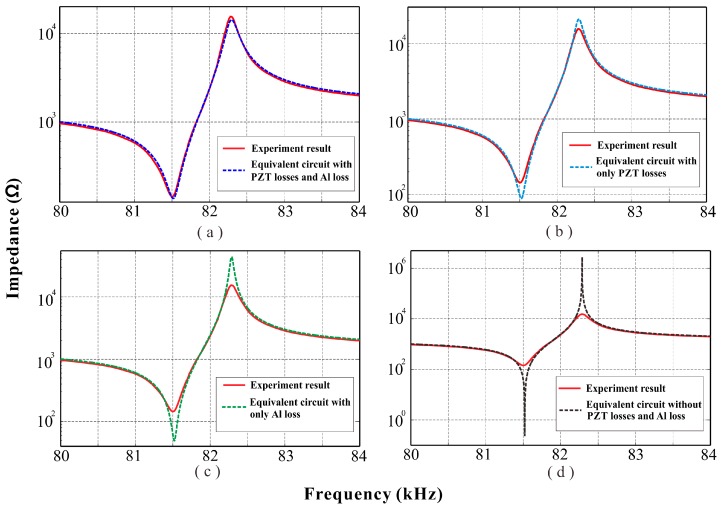
Comparison of impedance curves between experiment and equivalent circuit simulations: (**a**) experiment vs equivalent circuit with three PZT losses and aluminum loss (Al loss); (**b**) experiment vs equivalent circuit with three PZT losses; (**c**) experiment vs equivalent circuit with Al loss; (**d**) experiment vs equivalent circuit with no losses.

Figure 9 shows the vibrometer measurement results of this structure. The tested edge surface is perpendicular to the *x*-axis (Figure 1) and demonstrates longitudinal vibration at a frequency of 81.4 kHz. *A_or_* stands for the original cross-sectional area, while *A_c_* and *A_e_* are the contraction and extension of the original cross-sectional area, respectively. This structure was also measured using an Agilent 4294A impedance analyzer (Keysight Technologies, Inc., Santa Rosa, CA, USA); the frequency tested by the impedance analyzer was 81.5 kHz, which is close to the vibrometer result of 81.4 kHz. An impedance curve was obtained and compared with the equivalent circuit result. The geometry and material parameters, including loss factors, are listed in Table 1 and Table 2. For the loss factor of aluminum $tan\;\phi_{M}$, since the loss factor decreased with increasing frequency [33] and this structure was tested at high frequency (above 20 kHz), we assumed that it was equal to 1.0 × 10^-3^ [34,35]. The piezoelectric ceramic we used was Haiying P-51 (Haiying Enterprise Group Co., Ltd., Wuxi, Jiangsu, China), a PZT5 ceramic. Compared with PZT4, the loss factors in $tan\phi^{\prime }$, $tan\theta^{\prime }$, and $tan\phi_{P}$ of the P-51 are much higher; thus, the impact of losses can be clearly seen in the impedance curves in Figure 10. 

Figure 10a demonstrates that the impedance–frequency result of the equivalent circuit considering both the three PZT losses and the elastic loss of the metal substrate (aluminum loss or Al loss) has good agreement with the experimental result. It is notable that the deviations in Figure 10 are mostly the values of impedances *Z*_fR_ and *Z*_fA_ in resonance and antiresonance frequencies, respectively, while the frequency values simulated by the equivalent circuit have high accuracy and discrepancies in resonance and antiresonance frequencies are close to 0%. As for the values of impedances, for neglect of the aluminum loss (Figure 10b) or for neglect of the PZT losses (Figure 10c), the discrepancies in impedance values become higher compared with the experiment results. Moreover, the neglect of both the PZT losses and the aluminum loss leads to extremely high discrepancies (Figure 10d). The details are listed in Table 3. In summary, the consideration of losses (both PZT losses and Al loss) in the equivalent circuit increases its accuracy and thus makes it more effective in the study of piezoelectric structures.

## 5. Conclusions

This paper describes an equivalent circuit of a piezoelectric structure which consists of a slender aluminum beam and a PZT patch. The longitudinal vibration mode of this structure was studied, and the elastic loss of aluminum and three PZT losses were built into the equivalent circuit. The result of the equivalent circuit, namely, the impedance curve of this structure, has good agreement with the experiment result. The typical frequencies such as resonance and antiresonance simulated by the equivalent circuit are almost the same as the experiment results, while the values of impedances in resonance and antiresonance have percent errors of 4.3678% and 8.4631%, respectively. By contrast, neglect of the elastic loss of aluminum in the equivalent circuit leads to discrepancies of 37.890% and 34.149% in values of impedances, neglect of the PZT losses leads to discrepancies of 65.839% and 182.47%, and neglect of both the PZT losses and the aluminum loss leads to discrepancies of 99.831% and 18,105%. The introduction of PZT losses and aluminum loss to the equivalent circuit dramatically contributed to the result accuracy. This result can be extended to the analysis and design of piezoelectric sensors and actuators. In this paper, only longitudinal vibration is investigated; however, in practical applications, such as in multimode ultrasonic motors, bending or torsional modes are involved. Thus, the next step of this work is to study the equivalent circuits combined with multiple vibration modes on more practical assumptions, and to increase the result accuracy of equivalent circuits containing losses.

## Figures and Tables

**Table 1:** Loss factors of aluminum and PZT5.

Parameter	$tan\;\phi_{M}$	$tan\;\phi_{P}$	$tan\;\delta^{\prime }$	$tan\;\phi^{\prime }$	$tan\;\theta^{\prime }$
Value	1.000 × 10^−3^	10.20 × 10^−3^	2.000 × 10^−2^	11.70 × 10^−3^	21.80 × 10^−3^

**Table 2:** Geometry and material parameters of the piezoelectric structure.

Parameter	Value	Parameter	Value	Parameter	Value
*L*_0_ (mm)	12.60	*h_P_* (mm)	0.7000	$S_{11}^{E}$ (m^2^/N)	15.00 × 10^−12^
*L*_1_ (mm)	10.40	$\rho_{M}$ (kg/m^3^)	2700	*d*_31_ (C/N)	−185.0 × 10^−12^
*L_w_* (mm)	5.300	$\rho_{P}$ (kg/m^3^)	7450	$\varepsilon_{33}^{T}$	1750
*h_M_* (mm)	2.100	*Y_M_* (Gpa)	69.00	$C_{11}^{E}$ (N/m^2^)	15.00 × 10^10^

**Table 3:** Comparison of results between experiment and equivalent circuit (EC) simulations.

	f_R_ (Hz)	f_A_ (Hz)	Z_fR_ (Ω)	Z_fA_ (Ω)
Experiment	81,500	82,280	143.78	15,479
EC with PZT losses and Al loss	81,516	82,295	137.50	14,169
Percentage of error (%)	0.019632	0.018230	4.3678	8.4631
EC with only PZT losses	81,520	82,291	89.301	20,765
Percentage of error (%)	0.024540	0.013369	37.890	34.149
EC with only Al loss	81,521	82,287	49.116	43,724
Percentage of error (%)	0.025767	0.0085075	65.839	182.47
EC without PZT losses and Al loss	81,522	82,287	0.24246	2,817,880
Percentage of error (%)	0.026994	0.0085075	99.831	18,105

## Appendix A

The real part *R_P_*_1_ and imaginary part *G_P_*_1_ in the equivalent circuit of the PZT are

(A1) $$R_{P1}=\frac{A_{P}\left(K_{OA}\chi_{1}+K_{OB}\chi_{2}\right)}{\omega S_{11}^{E}\left(\chi_{1}^{2}+\chi_{2}^{2}\right)}$$

(A2) $$G_{P1}=\frac{A_{P}\left(K_{OB}\chi_{1}-K_{OA}\chi_{2}\right)}{\omega S_{11}^{E}\left(\chi_{1}^{2}+\chi_{2}^{2}\right)}$$

where *ω* is the angular frequency, *ω* = *2πf*, and *f* is frequency.

(A3) $$\delta_{1}=\frac{V_{P}C_{11}^{E}}{V_{P}+V_{M}}+\frac{V_{M}Y_{M}}{V_{P}+V_{M}}$$

(A4) $$\delta_{2}=\frac{V_{P}C_{11}^{E}\tan\phi_{P}}{V_{P}+V_{M}}+\frac{V_{M}Y_{M}\tan\phi_{M}}{V_{P}+V_{M}}$$

(A5) $$K_{OA}=\sqrt{\frac{\omega^{2}\rho_{B}\left(\sqrt{\delta_{1}^{2}+\delta_{2}^{2}}+\delta_{1}\right)}{2\left(\delta_{1}^{2}+\delta_{2}^{2}\right)}}$$

(A6) $$K_{OB}=\sqrt{\frac{\omega^{2}\rho_{B}\left(\sqrt{\delta_{1}^{2}+\delta_{2}^{2}}-\delta_{1}\right)}{2\left(\delta_{1}^{2}+\delta_{2}^{2}\right)}}$$

(A7) $$\chi_{1}=\tan\phi^{\prime }\sin(K_{OA}L_{0})\cosh(K_{OB}L_{0})-\cos(K_{OA}L_{0})\sinh(K_{OB}L_{0})$$

(A8) $$\chi_{2}=\tan\phi^{\prime }\cos(K_{OA}L_{0})\sinh(K_{OB}L_{0})+\sin(K_{OA}L_{0})\cosh(K_{OB}L_{0})$$

The real part *R_P_*_2_ and imaginary part *G_P_*_2_ in the equivalent circuit of PZT are

(A9) $$R_{P2}=\frac{A_{P}\left(\chi_{3}\chi_{5}+\chi_{4}\chi_{6}\right)}{\omega S_{11}^{E}\left(\chi_{5}^{2}+\chi_{6}^{2}\right)}$$

(A10) $$G_{P2}=\frac{A_{P}\left(\chi_{4}\chi_{5}-\chi_{3}\chi_{6}\right)}{\omega S_{11}^{E}\left(\chi_{5}^{2}+\chi_{6}^{2}\right)}$$

where

(A11) $$\chi_{3}=K_{OA}\tan\left(\frac{K_{OA}L_{0}}{2}\right)-K_{OB}\tanh\left(\frac{K_{OB}L_{0}}{2}\right)$$

(A12) $$\chi_{4}=K_{OA}\tanh\left(\frac{K_{OB}L_{0}}{2}\right)+K_{OB}\tan\left(\frac{K_{OA}L_{0}}{2}\right)$$

(A13) $$\chi_{5}=\tan\left(\frac{K_{OA}L_{0}}{2}\right)\tanh\left(\frac{K_{OB}L_{0}}{2}\right)+\tan\phi^{\prime }$$

(A14) $$\chi_{6}=1-\tan\phi^{\prime }\tan\left(\frac{K_{OA}L_{0}}{2}\right)\tanh\left(\frac{K_{OB}L_{0}}{2}\right)$$

and

(A15) $$C_{01}=\frac{L_{0}L_{w}}{h_{P}}\left[\varepsilon_{33}^{T}-\frac{d_{31}^{2}\left(1-\tan{\theta^{\prime }}^{2}+2\tan\theta^{\prime }\tan\phi^{\prime }\right)}{S_{11}^{E}\left(1+\tan{\phi^{\prime }}^{2}\right)}\right]$$

(A16) $$C_{02}=\frac{L_{0}L_{w}}{h_{P}}\left[-\frac{d_{31}^{2}\left(\tan\phi^{\prime }-\tan\phi^{\prime }\tan{\theta^{\prime }}^{2}-2\tan\theta^{\prime }\right)}{S_{11}^{E}\left(1+\tan{\phi^{\prime }}^{2}\right)}-\varepsilon_{33}^{T}\tan\delta^{\prime }\right].$$

The real part *R_M_*_1_ and imaginary part *G_M_*_1_ in the equivalent circuit of the metal substrate (Section 0) are

(A17) $$R_{M1}=\frac{A_{M}\left(\chi_{7}\chi_{9}+\chi_{8}\chi_{10}\right)}{\omega \left(\chi_{9}^{2}+\chi_{10}^{2}\right)}$$

(A18) $$G_{M1}=\frac{A_{M}\left(\chi_{8}\chi_{9}-\chi_{7}\chi_{10}\right)}{\omega \left(\chi_{9}^{2}+\chi_{10}^{2}\right)}$$

where

(A19) $$\chi_{7}=\delta_{1}K_{OA}-\delta_{2}K_{OB}$$

(A20) $$\chi_{8}=\delta_{1}K_{OB}+\delta_{2}K_{OA}$$

(A21) $$\chi_{9}=-\cos\left(K_{OA}L_{0}\right)\sinh\left(K_{OB}L_{0}\right)$$

(A22) $$\chi_{10}=\sin\left(K_{OA}L_{0}\right)\cosh\left(K_{OB}L_{0}\right).$$

The real part *R_M_*_2_ and imaginary part *G_M_*_2_ in the equivalent circuit of the metal substrate (Section 0) are

(A23) $$R_{M2}=\frac{A_{M}\left(\chi_{11}\chi_{13}+\chi_{12}\right)}{\omega \left(\chi_{13}^{2}+1\right)}$$

(A24) $$G_{M2}=\frac{A_{M}\left(\chi_{12}\chi_{13}-\chi_{11}\right)}{\omega \left(\chi_{13}^{2}+1\right)}$$

where

(A25) $$\chi_{11}=\left(\delta_{1}K_{OA}-\delta_{2}K_{OB}\right)\tan\left(\frac{K_{OA}L_{0}}{2}\right)-\left(\delta_{1}K_{OB}+\delta_{2}K_{OA}\right)\tanh\left(\frac{K_{OB}L_{0}}{2}\right)$$

(A26) $$\chi_{12}=\left(\delta_{1}K_{OA}-\delta_{2}K_{OB}\right)\tanh\left(\frac{K_{OB}L_{0}}{2}\right)+\left(\delta_{1}K_{OB}+\delta_{2}K_{OA}\right)\tan\left(\frac{K_{OA}L_{0}}{2}\right)$$

(A27) $$\chi_{13}=\tan\left(\frac{K_{OA}L_{0}}{2}\right)\tanh\left(\frac{K_{OB}L_{0}}{2}\right).$$

The real part *R_M_*_3_ and imaginary part *G_M_*_3_ in the equivalent circuit of the metal substrate (Sections 1 and 2) are

(A28) $$R_{M3}=\frac{A_{M}Y_{M}\left(\chi_{14}\chi_{16}+\chi_{15}\chi_{17}\right)}{\omega \left(\chi_{16}^{2}+\chi_{17}^{2}\right)}$$

(A29) $$G_{M3}=\frac{A_{M}Y_{M}\left(\chi_{15}\chi_{16}-\chi_{14}\chi_{17}\right)}{\omega \left(\chi_{16}^{2}+\chi_{17}^{2}\right)}$$

where

(A30) $$\chi_{14}=K_{OA}-K_{OB}\tan\phi_{E}$$

(A31) $$\chi_{15}=K_{OB}+K_{OA}\tan\phi_{E}$$

(A32) $$\chi_{16}=-\cos\left(K_{OA}L_{1}\right)\sinh\left(K_{OB}L_{1}\right)$$

(A33) $$\chi_{17}=\sin\left(K_{OA}L_{1}\right)\cosh\left(K_{OB}L_{1}\right).$$

The real part *R_M_*_4_ and imaginary part *G_M_*_4_ in the equivalent circuit of the metal substrate (Sections 1 and 2) are

(A34) $$R_{M4}=\frac{A_{M}Y_{M}\left(\chi_{18}\chi_{20}+\chi_{19}\right)}{\omega \left(\chi_{20}^{2}+1\right)}$$

(A35) $$G_{M4}=\frac{A_{M}Y_{M}\left(\chi_{19}\chi_{20}-\chi_{18}\right)}{\omega \left(\chi_{20}^{2}+1\right)}$$

where

(A36) $$\chi_{18}=\left(K_{OA}-K_{OB}\tan\phi_{E}\right)\tan\left(\frac{K_{OA}L_{1}}{2}\right)-\left(K_{OB}+K_{OA}\tan\phi_{E}\right)\tanh\left(\frac{K_{OB}L_{1}}{2}\right)$$

(A37) $$\chi_{19}=\left(K_{OA}-K_{OB}\tan\phi_{E}\right)\tanh\left(\frac{K_{OB}L_{1}}{2}\right)+\left(K_{OB}+K_{OA}\tan\phi_{E}\right)\tan\left(\frac{K_{OA}L_{1}}{2}\right)$$

(A38) $$\chi_{20}=\tan\left(\frac{K_{OA}L_{1}}{2}\right)\tanh\left(\frac{K_{OB}L_{1}}{2}\right).$$

## Appendix B

**Table A1:** Nomenclature.

Symbol	Meaning
*A_c_*	cross-sectional area of metal substrate in contraction situation
*A_e_*	cross-sectional area of metal substrate in extension situation
*α*	coefficient in displacement formula
*A_M_*	cross-sectional area of metal substrate
*A_or_*	cross-sectional area of metal substrate in original position
*A_P_*	cross-sectional area of PZT
*β*	coefficient in displacement formula
$C_{0}^{*}$	capacitor as a complex number
*C*_01_	real part of $C_{0}^{*}$
*C*_02_	imaginary part of $C_{0}^{*}$
$C_{11}^{E}$	stiffness under constant electric field
$C_{11}^{E*}$	stiffness under constant electric field as a complex number
$d_{31}$	piezoelectric constant
$d_{31}^{*}$	piezoelectric constant as a complex number
$D_{3}^{*}$	electric displacement of *z*-direction as a complex number
$\delta_{i}$(*i* = 1, 2)	coefficients in the formula of equivalent circuit
$e_{31}^{*}$	piezoelectric constant as a complex number
$\varepsilon_{33}^{T}$	dielectric constant under constant stress
$\varepsilon_{33}^{T*}$	dielectric constant under constant stress as a complex number
*E*_3_	electric field in *z*-direction
*f_A_*	antiresonance frequency
*f_R_*	resonance frequency
$F_{Mi}^{*}$ (*i* = 1, …, 6)	forces of metal substrate as a complex number
$F_{Pi}^{*}$ (*i* = 1, 2)	forces of PZT as a complex number
*G_Mi_* (*i* = 1, …, 4)	imaginary parts of complex numbers in equivalent circuit of metal substrate
*G_Pi_* (*i* = 1, 2)	imaginary parts of complex numbers in equivalent circuit of PZT
$h_{M}$	thickness of metal substrate
$h_{P}$	thickness of PZT
*I^*^*	current as a complex number
*j*	imaginary notation
$k_{0}^{*}$	wave number as a complex number
*K_OA_*	coefficients in the formula of equivalent circuit
*K_OB_*	coefficients in the formula of equivalent circuit
*L*_0_	length of Section 0
*L*_1_	length of Section 1 and Section 2
*L_w_*	width of piezoelectric structure
$m_{M}$	weight of metal substrate in Section 0
$m_{P}$	weight of PZT in Section 0
*N^*^*	force factor as a complex number
$\nu^{*}$	velocity as a complex number
$\nu_{Mi}^{*}$ (*i* = 1, …, 6)	velocity of metal substrate as a complex number
$\nu_{Pi}^{*}$ (*i* = 1, 2)	velocity of PZT as a complex number
*ω*	angular frequency
$\chi_{i}$ (*i* = 1, …, 20)	coefficients in the formula of equivalent circuit
*Q^*^*	charge of PZT electrode surface
$\rho_{B}$	density of composite structure
$\rho_{M}$	density of metal substrate
$\rho_{P}$	density of PZT
*R_Mi_* (*i* = 1, …, 4)	real parts of complex numbers in equivalent circuit of metal substrate
*R_Pi_* (*i*= 1, 2)	real parts of complex numbers in equivalent circuit of PZT
$S_{1}^{*}$	strain as a complex number
$S_{11}^{E}$	compliance under constant electric field
$S_{11}^{E*}$	compliance under constant electric field as a complex number
*t*	time
$\tan\delta^{\prime }$	“intensive” dielectric loss factor of PZT
$\tan\phi^{\prime }$	“intensive” elastic loss factor of PZT
$\tan\phi_{M}$	elastic loss factor of metal substrate
$\tan\phi_{P}$	“extensive” elastic loss factor of PZT
$\tan\theta^{\prime }$	“intensive” piezoelectric loss factor of PZT
*u^*^*	displacement along the *x*-direction
*U*	driving voltage
$U_{0}$	amplitude of driving voltage
$V_{M}$	volume of metal substrate in Section 0
$V_{P}$	volume of PZT
$X_{1}^{*}$	stress as a complex number
$X_{1M}^{*}$	stress of metal substrate as a complex number
$X_{1P}^{*}$	stress of PZT as a complex number
$Y_{B}^{*}$	composite Young’s modulus as a complex number
*Y_M_*	Young’s modulus of metal substrate
$Y_{M}^{*}$	Young’s modulus of metal substrate as a complex number
*Z*_0_	the expression of *C*_01_ in equivalent circuit
*Z*_1_	the expression of *C*_02_ in equivalent circuit
*Z_fA_*	impedance in antiresonance frequency
*Z_fR_*	impedance in resonance frequency
